# Cephalometric Characteristics of Various Ethnic Groups in Tanzania

**DOI:** 10.1155/ijod/6685596

**Published:** 2025-05-15

**Authors:** Ali Khamis Hamad, Ferdinand Mabula Machibya, Matilda M. Mlangwa, David N. Ngassapa

**Affiliations:** ^1^Department of Anatomy, School of Biomedical Sciences, Muhimbili University of Health and Allied Sciences, Dar es Salaam, Tanzania; ^2^Department of Orthodontics, Paedodontics and Community Dentistry, Muhimbili University of Health and Allied Sciences, Dar es Salaam, Tanzania

**Keywords:** cephalograms, craniofacial morphology, ethnic group, norms, Tanzania

## Abstract

**Objective:** The aim of this study is to investigate the craniofacial skeletal profile features of the Tanzanian population with normal occlusion using lateral skull cephalograms, to determine the differences in craniofacial characteristics among various Tanzanian ethnic groups, and to compare the cephalometric norms of Caucasians with those of Tanzanians.

**Materials and Methods:** Lateral cephalograms were taken from 142 male and 167 female Tanzanians, aged 18–30 years, all with acceptable and pleasing profiles and Class I occlusion, and no history of previous orthodontic treatments. Six linear and 14 angular cephalometric measurements were selected for analysis. A Student *t*-test was used to compare the mean cephalometric values between Tanzanians and Caucasians, while a one-way analysis of variance was applied to assess the inter-ethnic differences within the Tanzanian group.

**Results:** No significant differences were found between the Tanzanian ethnic groups, except for the ANB and NA-APog angles, which were increased in the Cushite group, and the Md1-ML angle, which was found to be reduced in the Bantu sample. Significant differences were observed between Tanzanians and Caucasians in all measurements (*p* < 0.05) except for the Wits appraisal in sagittal relationships and the S-Go:N-Me and SN-OcP measurements in vertical relationships.

**Conclusion:** The findings indicate that Tanzanian adults display distinct craniofacial characteristics, including a more convex facial profile, increased lower lip protrusion, dental proclination, and notable ethnic and intergroup variations.

## 1. Introduction

For thousands of years, researchers have attempted to classify human beings into various ethnic groups based on skin color, craniofacial features, and hair types [1]. However, such ethnic assessments are often inaccurate or inconsistent with how individuals identify themselves [2, 3]. The categories currently in use (Caucasian, Negroid, and Mongoloid) represent just a few of many classifications in which the size and shape of dental arches and/or facial skeletal patterns show considerable variation both within and among these groups [4]. For example, individuals classified as Negroid often exhibit characteristics of bimaxillary protrusion [5] and lower values for the interincisal angle [6]. Latin Americans have also been observed to show features of increased dental protrusion, with a skeletal pattern tending toward prognathism [7]. Since norms based on one specific population group can vary significantly when applied to other populations, researchers have recommended considering the ethnic differences when developing treatment plans for orthodontic patients [8].

Tanzania is home to an estimated 125 ethnic groups, which can primarily be classified into four groups: Bantu, Cushite, Nilo-Hamite, and San. The Bantu-speaking groups make up ~70%–80% of the population, while the Cushite groups account for about 5%–10%, and the Nilo-Hamites represent around 10%–15%. The San, typically comprising smaller populations, make up 1%–2% of the total population [9]. Notably, about 95% of Tanzanian ethnic groups are Bantu, with the exception of certain ethnic groups, such as the Alagwa (from the Kondoa district) [10], the Assa (from the Kiteto district) [11], the Burunge (from Kondoa) [12], the Datooga (from Karatu district) [13], the Gorowa (from Kondoa) [14], and the Kw'adza and Iraqw, which are categorized as Cushite [15]. Furthermore, the Barabaig and Maasai are Nilotic peoples residing in northern Tanzania [9]. As previous studies have reported variabilities in craniofacial morphology among different ethnic groups [16], and that norms established for one population should not be applied to other populations [17], it has become essential to determine the craniofacial norms for each specific ethnic group.

In cephalometric analysis, “normal values” refer to standardized measurements or reference points that reflect typical anatomical relationships within a specific population [18]. These values serve as important benchmarks for evaluating the skeletal and dental structures [7]. Typically, they are derived from large population studies that examine individuals without craniofacial anomalies, helping to establish the mean and range of various craniofacial parameters [19]. However, these values are not fixed and can vary depending on factors such as ethnicity, age, sex [20], and projection type. For example, 3D analysis offers more accurate representations of craniofacial anatomy, avoiding the projection distortions common in 2D analysis [21].

Previous research has established normal values using several criteria, including population-based studies, anthropometric measurements, age and sex considerations, growth patterns, and expert consensus [22]. While broader ethnic categories (macro groups) provide general reference values, these norms may not accurately reflect the craniofacial characteristics of smaller, specific populations (minor ethnic groups). Due to these variations, a tailored analysis is necessary to ensure accuracy [18]. Recognizing these differences is crucial for orthodontists and craniofacial surgeons to make informed diagnoses and develop appropriate treatment plans [23]. For instance, angular measurements such as the SNB and ANB angles may show different normative values for individuals of African or Asian descent compared to those of Caucasian descent [24].

The primary goal of our study is to investigate the craniofacial skeletal profiles of Tanzanian individuals with normal occlusion by analyzing lateral skull cephalograms. By identifying craniofacial features specific to various Tanzanian ethnic groups, we aim to provide a more detailed understanding of skeletal morphology variations within this population. This will allow clinicians to apply more precise, region-specific cephalometric norms, ultimately improving the quality of orthodontic care by tailoring treatment plans to the unique characteristics of each patient.

## 2. Materials and Methods

### 2.1. Sample Size Determination

This cross-sectional study was approved by the Muhimbili University Senate Research and Publications Committee (MUHAS-REC-05-2023-1654) in accordance with MUHAS research policies and Tanzania regulations governing human and animal subjects research. Written consent was obtained from each participant, who was fully informed of all procedures involved in the study. To determine the required sample size, the sample size formula for estimating a population mean was used:

(1) $$\begin{array}{c} n=Z^{2}\cdot \sigma^{2}/E^{2}, \end{array}$$

where *n* is the required sample size, *Z* is the Z score corresponding to the desired confidence level (for a 95% confidence level, *Z* = 1.96), *σ* is the estimated standard deviation of the population (5.4), and *E* is the margin of error (0.7) [25]. Based on this formula, the minimum sample size required for the study was calculated to be 229 participants.

In this study, a total of 309 volunteers (142 males and 167 females) were selected from the patient population attending the outpatient clinic at the Muhimbili University dental clinic in Dar es Salaam, Tanzania. All participants completed a questionnaire, confirming their Tanzanian citizenship by birth with both parents and grandparents being Tanzanians without interethnic marriage.

The participants met the inclusion criteria, which required having an acceptable and esthetically pleasing facial profile based on general standards of facial attractiveness [26], as assessed by a group of orthodontists. Each orthodontist independently evaluated the participants' facial esthetics through visual assessment. Additionally, participants were required to have Class 1 occlusion with no crowding and the presence of all teeth (excluding third molars). They also had no history of orthodontic treatment, craniofacial trauma, or related surgery. Participants with a history of jaw abnormalities, diseases, or syndromes were excluded from the study.

### 2.2. Data Collection Method

**Figure 1:**
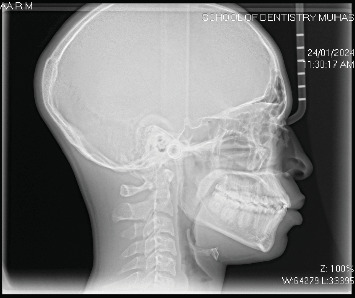
Lateral cephalometric radiograph.

**Figure 2:**
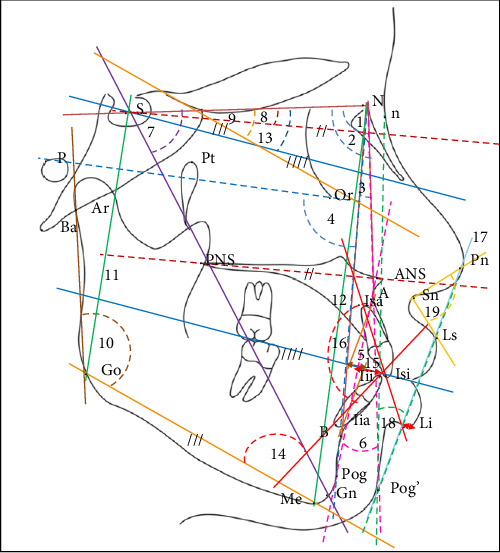
Landmarks, angular and linear variables used in this study. S, Sella; N, Nasion; Ar, Articular; A, Subspinale; B, Supramentale; Go, Gonion; Gn, Gnation; Pog, Pogonion; Or, Orbitale; ANS, anterior nasal spine; PNS, posterior nasal spine; lia, lower incisor root; lii, lower incisor crown; Isa, upper incisor root; Isi, upper incisor crown; Ls, upper lip; Me, Menton; P, Porion; Pn, pronasale; n, soft tissue nasion; Pog', soft tissue pogonion; Pt, Pterygoid; Ba, Basion; Li, lower lip; Sn, subnasale; //, ///, //// = parallel lines.

After a non-invasive direct extra and intraoral examination, a lateral cephalometric radiograph was obtained for each participant. All cephalograms were taken by the same operator, who has over 8 years of experience as a dental radiographer and has received relevant training in conducting cephalometric analyses, using Cone Beam Computed Tomography (X-VIEW 3D PAN CEPH, Trident, Italy). A calibration ruler was used to determine magnification, in compliance with standard radiation regulations [17]. All cephalometric images were acquired at a standardized magnification (1:1), and any necessary adjustments were made before analysis to ensure measurement consistency and accuracy. These adjustments followed established protocol to prevent distortion. Each digitalized image obtained was 41.45 cm × 34.68 cm in size and saved in JPG format (Figure 1). Cephalometric tracing was performed digitally by the same investigator, using Quick Ceph Studio, Version 5.2.6 (Quick Ceph Systems, Inc., FL 34236 US). To minimize fatigue-induced errors, the tracings were limited to 5–10 images per day. For the current analysis, 20 linear and angular measurements were selected, divided into four categories: sagittal, vertical, dentoalveolar relationships, and soft tissue profile. The assessment of sagittal basal relationships was based on the analyses by Steiner [7], Bailey, Proffit, and White [5, 6], Jacobson [27], and Downs [28]. The assessment of vertical basal relationship was adapted from the analyses of Brodie [29], Hasund [30], Bjork [31], Bailey, Proffit, and White [5, 6], and Jarabak [32]. The evaluation of dentoalveolar relationships followed the analyses of Bailey, Proffit, and White [5, 6] and Steiner [7]. The soft tissue assessment was based on the analyses of Ricket's [7], Holdaway's [33], and McNamara's [27]. All angular and linear measurements used are presented in Table 1 and Figure 2.

### 2.3. Reliability

A reliability test was conducted using the intraclass correlation coefficient (ICC). Forty lateral radiographs were randomly selected and remeasured twice, with a minimum 2-week interval between measurements.

### 2.4. Statistical Analysis

Kolmogorov–Smirnov test was used to assess the normality of the data for all variables. Statistical analysis was conducted to calculate the minimum, maximum, mean, and standard deviation. Since the sample showed a normal distribution, the difference between Tanzanian and established Caucasian cephalometric values was assessed using Student's *t*-test for independent samples. A one-way analysis of variance (ANOVA) at the 0.05 significance level was applied to evaluate the inter-ethnicity differences between Tanzanians. All data analyses were performed using RStudio Desktop for macOS 12+ (Posit Software, Boston, USA).

## 3. Results

The sample consisted of 309 lateral cephalograms from selected Tanzanian adults, with the majority belonging to Bantu ethnic group (90.61%) followed by Nilo-Hamites (7.12%) and Cushite (2.27%) ethnic groups. The mean age of the participants in the study was 27.59 ± 10.63 years. The mean age for females was 28.39 ± 11.78 years, while for males, it was 26.65 ± 9.05 years. The descriptive statistics for cephalometric values of the Tanzanian population are presented in Table 2, while the comparison between the mean cephalometric values is shown in Tables 3 and 4.

### 3.1. Reliability

The ICC indicated excellent reliability, with values ranging from 0.89 to 0.99 for all variables, and no significant variation was observed between the two measurements.

### 3.2. Comparisons of the Mean Values Between Tanzanian Ethnic Groups

Table 3 showed that the Cushite had significantly larger ANB and NA-APog angles (*p* < 0.05) compared to the Bantu and Nilo-Hamites groups regarding the sagittal basal relationship. No significant ethnic differences were observed in vertical basal relationships. In the dentoalveolar relationship, Bantu subjects displayed a significantly smaller Md1-ML angle (*p* < 0.05) compared to both Nilo-Hamite and Cushite subjects. However, no significant differences were found in soft tissue measurements, except for the H angle, which was significantly larger (*p* < 0.05) in Cushite subjects compared to the Bantu and Nilo-Hamites samples.

### 3.3. Comparisons Between Established Caucasian Norms and the Values of the Current Study

Table 4 shows that all mean values of the Tanzanian sample were statistically significantly different (*p* < 0.05) from the Caucasian means, except for the Wits, S-Go: N-Me, and SN-OcP angles, which showed no significant difference between Tanzanians and Caucasians.

Regarding the sagittal basal relationships, although the Tanzanian population exhibited a prognathic maxilla (SNA = 87.5°) and mandible (SNB = 83.22°) with a more forward position of the maxilla (Npog-FH = 91.51) compared to Caucasians, they still had a more convex facial profile (NA-Apog = 10.12°) with a Class II skeletal relationship due to a larger ANB value. In terms of vertical basal relationships, Tanzanians demonstrated a slightly downward and vertical growth direction of the mandible, as evidenced by greater SN-SGn angle, decreased posterior facial height (S-Go) and an increased ML-SNL angle. Furthermore, the N-Me distance (mean = 126.00°) and Ar–Go–Me angle (mean = 119.60°) indicated a reduced anterior vertical facial height compared to Caucasians, despite a decreased upper facial height (NL-SNL).

Dentally, the lower incisor was statistically significantly proclined (Md1-ML = 100.84) and protruded (Md1-APog = 8.21°), resulting in a reduced interincisal angle (Md1-Mx1 = 115.2°). In the soft tissue measurements, the lower lip was statistically significantly protruded (E-plane = 4.93°), with greater prominence of the upper lip (H angle = 18.68°), while a nasolabial angle (NLA = 81.25°) was less obtuse.

## 4. Discussion

Cephalometric analyses have been developed to determine norms for ideal facial proportions and occlusion, providing average measurements of dentoalveolar patterns and their respective ranges [34]. To diagnose malocclusion, cephalometric measurements are compared with standard values [35]. The small difference between the patient's measurement and the norm is interpreted as a normal variation, while the larger difference indicates structural deviation [36]. Variations within the normal range are considered harmonious, while variations outside this range are seen as incongruous and esthetically unpleasing [29]. Researchers such as Downs [28], Conner [37], Jacobson [27], and Tweed [7, 34] have established cephalometric values for their populations [38]. However, because of morphological differences between ethnic groups, their findings do not necessarily apply to other populations [39]. It is unscientific to apply the standards of one ethnic group to another or to apply the standards of one subgroup to another [40]. This has motivated other researchers to investigate cephalometric norms in various populations, including Sudanese [6], Chinese [41], Central Indian [42], North Indian [16], Mewari [35], Coastal Andhra [43], Japanese [44], Maharashtrian [28], and others.

The results of the current study align with many other studies comparing European-origin populations with other ethnic groups. These studies have shown that each racial group has its own standards, and even within the same population, different subgroups may have distinct norms [40]. Regarding sagittal basal relationships, the Tanzanian population exhibited increased mean values for the SNA, SNB, ANB, NPog-FH, and NA-APog angles, indicating a prognathic maxilla and mandible, as well as a more convex facial profile with a Class II skeletal relationship compared to Caucasians. Our results are consistent with Pereira's findings in the Portuguese sample [45] and Huang's analysis of dentofacial pattern in the Birmingham population, which showed that African Americans had higher means for SNA, ANB, and ANB angular measurements, with more convex profiles and bimaxillary protrusive relative to skull base [46].

In terms of vertical basal relationship, all parameters in the Tanzanian population were greater than that of the Caucasians, except for the S-Go: N-Me and SN-OcP measurements, which were within the established standard values. However, our preferred vertical measurements were the S-Go: N-Me and Ar–Go–Me, as both seemed to offer more practical value due to lower error rates. The S-Go: N-Me measurement depends on four reference points, reducing the likelihood of errors. Likewise, the S-Go and N-Me linear measurements are taken in the vertical plane. The Ar–Go–Me angle is related to the mandible, which plays a significant role in facial vertical growth. This anatomical component is a more reliable parameter than structures that depend on the SN line, such as the NL-NSL, SN-OcP, ML-NSL, and SN-SGn angles, which are farther from the cranial base.

For dental analysis, we focused on the Md1-ML, Md1-APog and Md1-Mx1 parameters. The inclination and position of the lower incisors were emphasized, as they are a key focus in modern orthodontic treatments [47]. The position of the upper incisors can be evaluated indirectly through the interincisal angle and overjet. Additionally, only the anterior teeth were considered in the analysis, as they are more directly related to facial esthetics, which is often the primary concern of orthodontic patients.

Regarding soft tissue analysis, Tanzanian subjects exhibited both upper and lower lip protrusions when compared to Caucasian norms, particularly in terms of the H angle and NLA for the upper lip and the E-plane for the lower lip. These findings align with studies showing the influence of maxillary and mandibular incisors' position and inclination on lip prominence [48]. The mean value of the H angle was greater for Tanzanians than for Holdaway's standards. Additionally, the lower lip in Tanzanian subjects was positioned more anteriorly relative to the E-line than the norm established by Ricketts. These results are similar to those reported for South Indian [49], Saudi [33], and Bangladeshi [3, 50] populations, though greater than those observed in the Mexican population [7, 25]. An increased linear measurement between the lower lip and the H line may indicate a retruded lip or prominent chin. However, inter-ethnic differences in soft tissue measurements within the Tanzanian sample were not evident, except for the H angle, which was significantly larger in the Cushite group compared to the Nilo-Hamites and Bantu groups.

## 5. Conclusions

This study clearly demonstrates that most of the cephalometric mean values for the Tanzanian population differ from the established Caucasian values, indicating craniofacial morphological variation between the Tanzanian and Caucasian populations. The following differences were observed in the Tanzanians compared to Caucasians:

• The Tanzanian population exhibits bimaxillary protrusion, with a normal relationship between maxilla and mandible, a convex facial profile, and an increased vertical dentoalveolar dimension.
• Our findings also indicate that Tanzanians have reduced upper facial height, increased lower facial height, decreased anterior and posterior facial height, a normal ratio of posterior to anterior facial height, a normal vertical dentoalveolar dimension, and protrusive lips.
• The results of this study reinforce the importance of not applying the specific norms of one group to another population.

## Figures and Tables

**Table 1:** Hard and soft tissue variables.

Variables	Interpretation
Sagittal basal relationships	
SNA (deg)	Maxillary position relative to the cranial base
SNB (deg)	Mandibular position relative to the cranial base
ANB (deg)	Maxillary and mandibular position relative to each other
NPog-FH (deg)	Degree of retrusion and protrusion of the mandible
Wits (mm)	Maxillary and mandibular position relative to the occlusal plane
NA-APog (deg)	Maxillary and mandibular position relative to the most anterior part of the cranial base
Vertical basal relationships	
SN-SGn (deg)	Estimation of mandibular growth direction
NL-NSL (deg)	Maxillary cant relative to the cranial base
ML-NSL (deg)	Mandibular cant relative to the cranial base
Ar–Go–Me (deg)	Estimation of vertical facial height
S-Go (mm)	Length of posterior face height
N-Me (mm)	Length of anterior face height
S-Go: N-Me	Facial ratio
SN-OcP (deg)	Vertical dentoalveolar dimension
Dentoalveolar relationship	
Md1-ML (deg)	Proclination of incisors in the mandible
Md1-APog (mm)	Position of lower incisors relative to the upper and lower jaw
Md1-Mx1 (deg)	The inclination of lower and upper incisors to each other
Soft tissue profile	
E Plane (mm)	Protrusion of the lower lip relative to the esthetic line
H angle (deg)	Prominence of the upper lip to N′pog' line
NLA (deg)	Upper lip protrusion

**Table 2:** Descriptive statistics for cephalometric values of Tanzanian populations.

Variables	Mean	±SD	SE	Minimum	Maximum	95% CI	
						Lower	Upper
Sagittal basal relationships							
SNA	87.50	4.12	0.23	75.60	98.40	87.09	88.01
SNB	83.22	4.11	0.23	71.20	93.50	82.76	83.68
ANB	4.33	2.32	0.13	−2.40	9.70	4.07	4.59
NPog-FH	91.51	3.28	0.18	81.70	102.30	91.14	91.87
Wits	−1.07	3.34	0.19	−10.40	8.10	−1.44	−0.69
NA-APog	10.12	5.32	0.30	−5.30	22.30	9.52	10.71
Vertical basal relationships							
SN-SGn	66.74	4.03	0.23	55.90	81.00	66.29	67.19
NL-NSL	5.391	3.54	0.20	−4.60	18.40	4.99	5.79
ML-NSL	32.52	6.59	0.38	16.00	51.30	31.79	33.26
Ar–Go–Me	119.6	7.76	0.44	99.3	140.1	118.77	120.51
S-Go	80.53	7.47	0.42	59.30	102.50	79.69	81.36
N-Me	126.0	7.94	0.45	109.2	148.7	125.16	126.93
S-Go: N-Me	63.94	5.12	0.29	52.00	76.40	63.37	64.51
SN-OcP	14.08	5.03	0.29	−0.40	29.70	13.51	14.64
Dentoalveolar relationship							
Md1-ML	100.84	6.95	0.40	81.1	119.1	100.06	101.62
Md1-APog	8.21	2.70	0.15	−0.10	13.70	7.91	8.51
Md1-Mx1	115.2	9.07	0.51	90.6	136.5	114.18	116.22
Soft tissue profile							
E-Plane	4.932	3.08	0.18	−2.10	15.70	4.59	5.28
H angle	18.68	3.40	0.19	8.40	27.20	18.29	19.06
NLA	81.25	12.96	0.74	44.70	115.70	79.79	82.69

Abbreviations: SD, standard deviation; SE, standard error.

**Table 3:** Comparisons of the mean values between Tanzanian ethnic groups.

Variables	Bantu		Nilo-Hamites		Cushite		Significances	
	Mean	±SD	Mean	±SD	Mean	±SD	*f*-values	*p*-value
Sagittal basal relationships								
SNA	87.38	4.15	89.85	3.66	87.27	2.04	2.824	0.094
SNB	83.13	4.04	85.06	4.49	81.21	4.55	0.201	0.654
ANB	4.25	2.25	4.79	2.77	6.05	3.29	4.771	0.029^a^
NPog-FH	91.52	3.14	91.79	3.91	90.10	6.05	0.358	0.55
Wits	−1.19	3.35	0.38	3.04	−0.73	3.32	2.647	0.105
NA-APog	9.92	5.17	11.52	6.09	13.53	7.76	4.833	0.028^a^
Vertical basal relationships								
SN-SGn	66.81	3.93	65.21	4.44	68.53	5.72	0.098	0.755
NL-NSL	5.46	3.48	3.61	3.67	8.12	3.39	0.001	0.974
ML-NSL	32.72	6.33	29.50	8.62	34.13	8.37	0.969	0.326
Ar–Go–Me	119.83	7.48	117.07	10.06	120.07	10.44	0.929	0.336
S-Go	80.29	7.49	83.89	7.21	79.47	5.42	1.378	0.241
N-Me	126.06	8.21	125.90	4.64	126.09	4.71	0.002	0.963
S-Go: N-Me	63.75	5.03	66.67	5.83	63.06	4.31	1.905	0.168
SN-OcP	14.21	4.89	11.31	5.42	17.36	6.41	0.193	0.661
Dentoalveolar relationship								
Md1-ML	100.54	6.80	103.63	8.75	103.94	3.39	5.046	0.025^a^
Md1-APog	8.21	2.66	8.14	2.97	8.70	3.86	0.082	0.774
Md1-Mx1	115.49	9.00	111.89	9.79	113.71	8.55	2.328	0.128
Soft tissue profile								
E-Plane	4.88	3.12	5.28	2.68	5.90	3.03	1.027	0.312
H angle	18.57	3.39	19.29	2.96	21.17	4.04	4.471	0.035^a^
NLA	81.53	12.79	77.21	13.75	82.57	16.79	0.642	0.424

Abbreviation: SD, standard deviation.

^a^Significance level at *p* < 0.05.

**Table 4:** Comparison between established Caucasian norms and the values of the current study.

Variables	Caucasian norms		Tanzanian				
	Values	Analysis	Mean	±SD	SE	*t*-value	*p*-value
Sagittal relationships							
SNA	82	Steiner	87.50	4.12	0.23	23.678	^a^
SNB	79	Bailey	83.22	4.11	0.23	18.050	^a^
ANB	3	Bailey	4.33	2.32	0.13	10.087	^a^
NPog-FH	85	Bailey	91.51	3.28	0.18	34.877	^a^
Wits	−1	Jacobson	−1.07	3.34	0.19	0.007	0.994
NA-APog	3	Downs	10.12	5.32	0.30	23.513	^a^
Vertical relationships							
SN-SGn	66	Brodie	66.74	4.03	0.23	3.216	^b^
NL-NSL	8	Hasund	5.391	3.54	0.20	−12.955	^a^
ML-NSL	29	Hasund	32.52	6.59	0.38	9.394	^a^
Ar–Go–Me	124	Bjork	119.6	7.76	0.44	−9.874	^a^
S-Go	88	Bailey	80.53	7.47	0.42	−17.594	^a^
N-Me	137	Bailey	126.0	7.94	0.45	−24.267	^a^
S-Go:N-Me	62–65	Jarabak	63.94	5.12	0.29	0.002	0.999
SN-OcP	14	Bailey	14.08	5.03	0.29	0.268	0.789
Dentoalveolar relationship							
Md1-ML	96	Bailey	100.84	6.95	0.40	12.245	^a^
Md1-APog	3	Bailey	8.21	2.70	0.15	33.883	^a^
Md1-Mx1	130	Steiner	115.2	9.07	0.51	−28.674	^a^
Soft tissue profile							
E-Plane	−2	Ricket	4.93	3.08	0.18	39.546	^a^
H angle	15	Holdaway	18.68	3.40	0.19	19.024	^a^
NLA	102	McNamara	81.25	12.96	0.74	−28.154	^a^

Abbreviations: SD, standard deviation; SE, standard error.

^a^ *p* < 0.0001.

^b^ *p* < 0.001.

## Data Availability

The data that support the findings of this study are available on request from the corresponding author. The data are not publicly available due to privacy or ethical restrictions.
